# Impact of serotype and sequence type on the preferential aerosolization of *Streptococcus suis*

**DOI:** 10.1186/s13104-016-2073-8

**Published:** 2016-05-14

**Authors:** Léa Gauthier-Levesque, Laetitia Bonifait, Nathalie Turgeon, Marc Veillette, Phillipa Perrott, Daniel Grenier, Caroline Duchaine

**Affiliations:** Centre de Recherche de l’Institut Universitaire de Cardiologie et de Pneumologie de Québec (CRIUCPQ), 2725 Chemin Sainte-Foy, Quebec City, QC Canada; Centre de Recherche en Infectiologie Porcine et Avicole (CRIPA), Fonds de Recherche Nature et Technologies du Québec (FRQNT), Saint-Hyacinthe, QC Canada; Groupe de Recherche en Écologie Buccale (GREB), Faculté de Médecine Dentaire, Université Laval, Quebec City, QC Canada; Département de Biochimie, Microbiologie et Bio-informatique, Faculté des Sciences et de Génie, Université Laval, Quebec City, QC Canada

**Keywords:** Preferential aerosolization, *Streptococcus suis*, Serotype 2, Sequence type, Airborne

## Abstract

**Background:**

*Streptococcus suis* is a swine pathogen that causes pneumonia, septicemia and meningitis. It is also an important zoonotic agent responsible of several outbreaks in China. *S. suis* strains are classified into 35 serotypes based on the composition of their polysaccharide capsule. *S. suis* serotype 2 causes the majority of severe infections in pigs and in human, and can be further subdivided into sequence types (STs) based on multilocus sequence typing. The ST1 is associated with highly virulent strains. In North America, the strains most commonly isolated belong to ST25 and ST28, which are respectively moderately and weakly virulent in a mouse model. The presence of *S. suis* bioaerosols in the air of swine confinement buildings has been previously demonstrated. The aim of this study was to better understand the aerosolization behaviour of *S. suis* by investigating the preferential aerosolization of various strains of *S. suis*, belonging to different serotypes or STs, using in-house developed environmental chamber and bubble-burst nebulizer. qPCR technology was used to analyze the ratio of *S. suis* strains.

**Results:**

The results suggest that the highly virulent serotype 2 ST1 strains are preferentially aerosolized and that the *S. suis* preferential aerosolization is a strain-dependent process.

**Conclusion:**

These observations will need to be confirmed using a larger number of strains. This study is a proof of concept and increases our knowledge on the potential aerosol transmission of *S. suis.*

## Background

*Streptococcus suis* is a swine pathogen that causes important economic losses in the swine industry worldwide. Swine are natural hosts of *S. suis,* which can be isolated from their tonsils and nasal cavities, as well as genital and digestive tracts [1, 2]. *S. suis* causes a wide range of illness in swine such as meningitis, septicemia, pneumonia, endocarditis and arthritis. It is also known as an important zoonotic agent for individuals in close contact with pigs or pork by-products [3]. Two serotype 2 human infection outbreaks occurred in China with more than 200 cases declared and 50 deaths reported [4]. However, in North America and Europe, human *S. suis* infections are still considered sporadic. Seven human deaths related to *S. suis* infections have been described in Canada and the United States since 1991 [5–11].

*Streptococcus suis* strains are classified into 35 different serotypes on the basis of the antigenicity of their capsular polysaccharide [12–15]. Amongst these serotypes, serotype 2 is the most commonly isolated from diseased animals [1, 3, 12, 16, 17]. Multilocus sequence typing (MLST) allowed separating the serotype 2 strains into 16 sequence types (STs) based on genetic variations. The ST1 is associated with invasive and highly virulent strains [18–20]. In North America, strains belonging to ST25 and ST28 are most often recovered in infected pigs [21]. They are respectively strains with moderate and weak virulence in a mouse model [21].

Healthy carrier pigs could act as an infectious reservoir for pathogenic strains of *S. suis*. Many modes of transmission have been proposed for the transfer of *S. suis* between swine within the herd. The most accepted relates to a transmission of *S. suis* by a ‘‘nose-to-nose’’ contact between uninfected and infected pigs, especially when animals show clinical signs of infection [1, 22]. In 2001, Berthelot-Hérault et al. [22, 23] first emphasized the transmission of *S. suis* virulent serotype 2 strains through aerosols from infected swine to pathogen-free swine. Then, Dekker et al. [24] further supported these observations and showed that a clinical serotype 9 strain could be transmitted through aerosols. Very recently, Bonifait et al. [25] demonstrated the presence of *S. suis*, more particularly serotype 2, in bioaerosols of swine confinement buildings (SCBs), with and without recent documented infection cases. All the above studies support the potential of air transmission of this swine and zoonotic pathogen.

The notion of preferential aerosolization was introduced by Parker et al. [26] when they showed that *Mycobacterium intracellulare* was more concentrated than *Mycobacterium scrofulaceum* in air samples produced by an equally mixed solution using smooth bubble-burst nebulizer. Moletta et al. [27] have also highlighted this concept of preferential aerosolization of some microorganisms in anaerobic microbial communities and consequently suggested that aerosolization appears to be a non-randomly phenomenon and that some bacteria are more prone to be transferred to the air.

The aim of this study was to investigate the aerosolization behaviour of *S. suis* in a controlled environment. In this regard, the possibility of a preferential aerosolization of different isolates of *S. suis* has been studied in terms of serotype (serotype 2, serotype 5) and of ST (ST1, ST25, ST28) in order to determine whether the virulence of the strains may be related to their aerosolization.

## Methods

### Serotype and sequence type (ST)

The serotype 2 *S. suis* S735 was aerosolized with either strain of serotype 5 (Amy12C, 4B) included in this study, in order to compare the preferential aerosolization of *S. suis* serotype 2. The choice of serotype 5 strains was based on the fact they present differences in morphological characteristics. Indeed, *S. suis* serotype 5 appears to possess a capsule thinner than that of *S. suis* serotype 2 [28]. Seven strains belonging to either ST1, ST25 or ST28 was tested (Table 1) [21]. Each strain was nebulized separately with *S. suis* Amy12C (serotype 5) used as an internal reference strain. Strains of *S. suis* used in this study are listed in Table 1. Bacteria were grown in Todd Hewitt Broth (THB) (Difco Laboratories, Becton, Dickinson and Company, Sparks, MD, USA.) at 37 °C.

**Table 1:** Serotype, sequence type (ST), origin and diseases of *S. suis* strains used in this study

Strain	Serotype	ST	Origin	Tissue/disease
P 1/7	2	1	United Kingdom	Meningitis
S735	2	1	Netherlands	Pneumonia
MGGUS2	2	1	United States	Brain
MGGUS4	2	25	United States	Septicaemia
MNCM51	2	25	Thailand	Septicaemia
MGGUS10	2	28	United States	Lung
MGGUS11	2	28	United States	Lung
Amy12C	5	NA	Canada	Infected pig
4B	5	NA	Canada	Infected pig

### Cell surface hydrophobicity test

Cell surface hydrophobicity was determined by measuring the adsorption of *S. suis* cells to n-hexadecane as described previously [29]. Assays were performed in triplicate.

### Experimental chamber

**Fig. 1:**
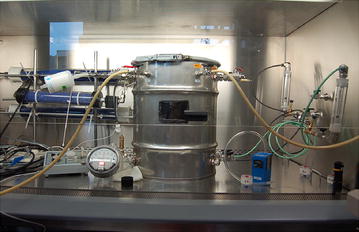
Experimental chamber used for the preferential aerosolization process

The chamber used in this study was designed, built and characterized by Perrott et al. (Manuscript in preparation). This chamber consists in a stainless steel drum of 45.2 L, was 470 mm in height by 350 mm in diameter and was airtight by a lid (Fig. 1). Ports were created to allow nebulization or air sampling.

### Preparation of nebulizing solutions

For each comparative analysis, two strains of *S. suis* were grown separately in 300 mL of THB overnight, and harvested by centrifugation (10 min at 7000×*g)*. Cells were washed twice with 1X Phosphate Buffered Saline (PBS, Lonza, Bâle, Switzerland) and suspended in PBS at an optical density 660 nm = 1.5 (about 4 × 10^8^ bacteria/mL) using a GeneQuant pro spectrometer (model 80-2114-98, GE Healthcare Biosciences, Buckinghamshire, England). Nebulizing solutions of 150 mL were prepared and contained both strains of *S. suis*.

### Preferential aerosolization assay

For the preferential aerosolization assays, two different *S. suis* strains were mixed in the nebulizing solution. Ratios in the nebulizing solution and in the air were compared to evaluate if one of the two strains was enriched in the bioaerosols compared with the original bacterial suspension. Aerosolization assays of tested strains were performed in duplicate.

### Nebulization

**Fig. 2:**
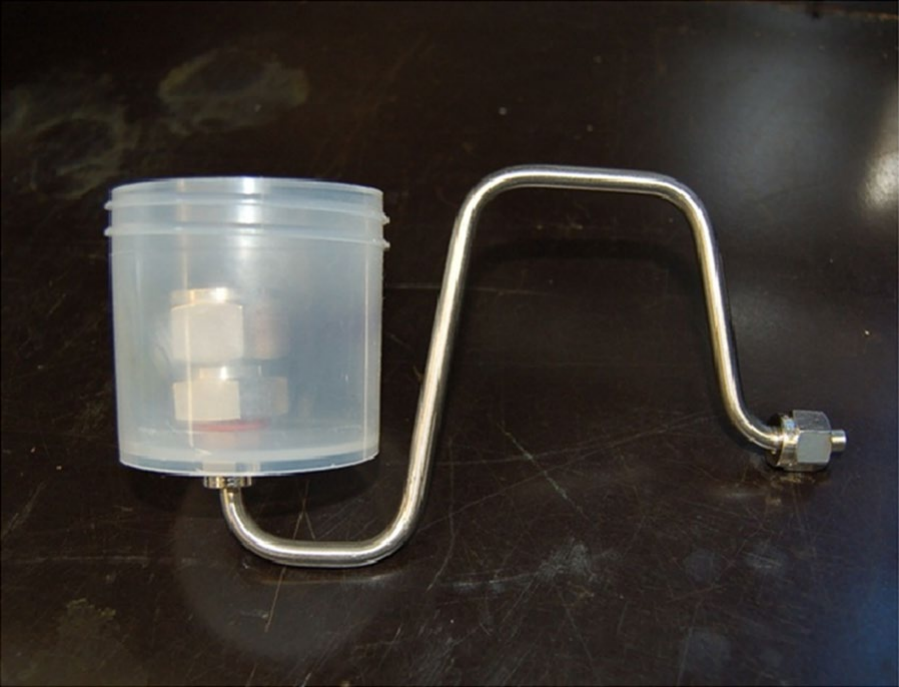
Smooth bubble-burst nebulizer used for the preferential aerosolization process

The nebulizer used in this study has been described by Perrot et al. (Manuscript in preparation). Briefly, this nebulizer was designed to mimic the bubbling process and minimize the stress associated with the aerosolization of bacteria. Using commercial nebulizer, as jet nebulizer, a high-speed airstream hits the liquid bacteria suspension and creates cell wall damages [30–32]. The bubbling nebulizer was located inside the experimental chamber. It was made with a 250 mL of polypropylene container to allow sterilization by an autoclave (Fig. 2). At the bottom of the container, a hole was made to fix a stainless steel tube that allows air to pass. At the end of the tube, the air passes through a cotton fabric that makes bubbles and creates bioaerosols.

### Aerosolization

Aerosolization was performed with a nebulization set at 4 L/min and an addition of dilution air at 6 L/min. Prior to bacterial aerosolization, PBS was aerosolized for conditioning the chamber; this was followed by a purge of the chamber prior to the baseline air sampling. Bacteria were first nebulized during 20 min to fill the chamber with aerosols and then, the air of the chamber was sampled. Between each duplicate, the nebulizer and the nebulizing solution were changed. The temperature and relative humidity were monitored using the Omega RH-USB probe (Omega Engineering, Inc., Stamford, CT). Introducing dry air into the chamber controlled the temperature and humidity. The humidity was also controlled using silica beads.

### Air sampling

Aerosols and baselines were sampled using SKC 37 mm cassettes (SKC Inc., Eighty-Four, PA, USA) loaded with a 0.8 μm polycarbonate filter (SKC Inc., Eighty-Four, PA, U.S.A.). The cassettes were connected to a Gilian AirCon2 High Volume Air Sampler (Gilian Instrument Corp., W. Caldwell, NJ, USA.) set at 10 L/min, for 30 min. Aerosol samples were taken during the nebulization. Filter samples were eluted in 5 mL of PBS using Genie-2 vortex (Scientific industries, Bohemia, NY, USA.) for 15 min. A blank was prepared for each experiment (cassettes not plugged to a pump).

### DNA extraction

Aliquots of air samples and nebulizing solution (1.5 mL) were centrifuged for 10 min at 14,000×*g* and the pellets were stored at −20 °C until use. DNA extraction was performed with MOBio PowerLyser^®^ UltraClean^®^ Microbial DNA kit (Carlsbad, CA, USA.) following the manufacturer’s instructions using homogenization with Mixer Mill MM301 (Retsch, Düsseldorf, Germany) at 20 movements per min during 10 min. DNA was eluted with 50 μL of elution buffer supplied with the kit. Samples were kept at −20 °C.

### Quantitative PCR

Quantitative PCR (qPCR) analyses were performed with the Bio-Rad CFX 96 Touch™ Real-time PCR Detection System (Bio-Rab Laboratories, Mississauga, ON, Canada). Primers and probes were purchased from Integrated DNA Technologies (Coralville, IA, USA.) and are listed in Table 2. Results were analyzed using the CFX Manager™ Software version 3.1 (Bio-Rad Laboratories). Quantification of *S. suis* serotype 2 was performed according to Nga et al. [33]. Primers target the *cps2J* gene that is part of the serotype 2 capsular polysaccharide operon [34]. As standard curve, a 10-fold dilution of *S. suis* S735 genomic DNA was used. Quantification of *S. suis* serotype 5 was made as previously described by Wang et al. [35]. Primers target the *cps5I* gene that is part of the serotype 5 capsular polysaccharide operon coding for a glycosyltransferase [35]. As standard curve, a 10-fold dilution of *S. suis* 4B genomic DNA was used.

**Table 2:** Primers and probe used in this study

Primer/probe	Target	Sequence	Amplicon length (pb)	Reference
CpS2Jf	*cps2J*	5′-GGTTACTTGCTACTTTTGATGGAAATT-3′	88	Nga et al. [33]
CpS2Jr	*cps2J*	5′-CGCACCTCTTTTATCTCTTCCAA-3′	88	Nga et al. [33]
CpS2Jp	*cps2J*	5′-FAM-TCAAGAATCTGAGCTGCAAAAG TGTCAAATTGA-TAMRA-3′	88	Nga et al. [33]
CpS5If	*cps5I*	5′-TTTTCGTTGTATTTTCCAAA-3′	262	Wang et al. [35]
CpS5Ir	*cps5I*	5′-TCCAAACATTATCCCCTATT-3′	262	Wang et al. [35]

*NA* Not applicable

## Results

### Temperature and relative humidity

The temperature and the relative humidity inside the chamber during the preferential aerosolization assays were respectively 22.9 °C and 63.5 %.

### Cell surface hydrophobicity

Table 3 reports the relative cell surface hydrophobicity for strains of *S. suis* used in this study. All *S. suis* serotype 2 strains showed a low cell surface hydrophobicity (≤11 %) compared to the high hydrophobicity observed for the serotype 5 strains (≥87 %).

**Table 3:** Relative cell surface hydrophobicity of *S. suis* strains used in this study

Serotype	ST	Strains of *S. suis*	Hydrophobicity (%)
2	1	P1/7	11 ± 5
		S735	6 ± 7
		MGGUS2	1^a^
	25	MGGUS4	5 ± 2
		MNCM51	5 ± 1
	28	MGGUS10	>0^b^
		MGGUS11	5 ± 2
5	NA	Amy12C	88 ± 7
		4B	87 ± 10

*NA* Not applicable

^a^Two out of three values were negative

^b^The three values were negative

### Preferential aerosolization assay

**Fig. 3:**
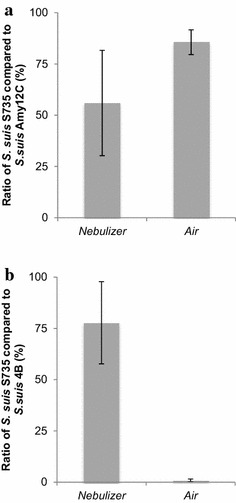
Comparative analysis of strains belonging to different serotypes. **a** Ratio of *S. suis* S735 (serotype 2) in the nebulizing solution and in the air compared to *S. suis* Amy12C (serotype 5) and **b** ratio of *S. suis* S735 (serotype 2) in the nebulizing solution and in the air compared to *S. suis* 4B (serotype 5)

The ratios of the tested strains of *S. suis* in the nebulizing solution and the bioaerosols are reported in Fig. 3. A ratio of 100 % means that the tested strain represents the entire sample. The serotype 2 strain *S. suis* S735 was aerosolised along with either *S. suis* Amy12C or *S. suis* 4B, two serotype 5 isolates. Figure 3a shows that *S. suis* S735 is preferentially recovered in bioaerosols when aerosolized concomitantly with *S. suis* Amy12C. However, although the ratio in the air is higher than that in the nebulizing solution, these differences are not statistically significant (*p* < 0.05). Regarding *S. suis* 4B, an opposite result was obtained. Indeed, the serotype 5 strain was the one almost exclusively detected in the bioaerosols (Fig. 3B).

**Fig. 4:**
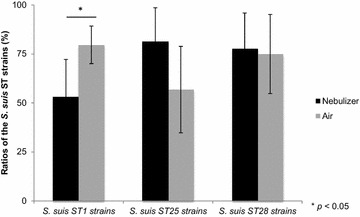
Ratio of different ST of *S. suis* serotype 2 in the nebulizing solution and in the air. ST1, 3 strains tested. ST25, 2 strains tested. ST28, 2 strains tested. A Student’s *t* test analysis for normal distribution was used to perform comparisons. * Significantly different at *P* value <0.05

Strains belonging to different STs were aerosolized separately with *S. suis* Amy12C (serotype 5) used as an internal reference strain. The ST1 virulent strains selected were isolated from pigs meningitis/septicemia cases [21]. ST25 and ST28 strains were isolated from pigs septicemia and pneumonia cases, respectively [21]. Figure 4 compares the aerosolization of the *S. suis* ST1 (3 strains), ST25 (2 strains) and ST28 (2 strains) when pooling data obtained for the different strains. For *S. suis* ST1, the ratio in the nebulizing solution was 53 %, while the ratio in the air was 80 % which is statistically different (*p* = 0.0059). The ratio in the nebulizing solution for *S. suis* ST25 was 81 % and its ratio in the air was 57 %. Lastly, ratios in the nebulizing solution and in the air for the *S. suis* ST28 were respectively 78 and 75 %. There are no statistical differences for the *S. suis* ST25 and ST28.

## Discussion

The presence of *S. suis* in the air of SCBs and the potential risks of transmission of this pathogen through bioaerosols have been previously studied [22, 24, 25]. However a better understanding of the aerosolization process of *S. suis* is essential and was the topic of this study. More specifically, we proposed the hypothesis of a preferential aerosolization of *S. suis* in function of serotype and ST.

Given that *S. suis* serotype 2 is the serotype most commonly isolated from diseased animals, it can be suggested that this serotype is preferentially aerosolized compared to the others. When comparing the preferential aerosolization behaviours of *S. suis* S735 (serotype 2) with *S. suis* Amy12C or *S. suis* 4B (serotype 5), it appears that aerosolization is more likely to be strain-dependent than serotype-dependent, although additional strains belonging to different serotypes should be tested. Both strains of *S. suis* serotype 5 possessed characteristics (thin capsule and high hydrophobicity) that differentiate them from serotype 2 strains [28]. Consequently, these two properties appear not to have significant impact on the preferential aerosolization of *S. suis*.

Preferential aerosolization occurs when the ratio of bacteria is higher in the air compared to the original source. In this regard, *S. suis* ST1 strains tested in this study, but not *S. suis* ST25 and ST28 strains, appear to be statistically preferentially aerosolized. Differences at the gene and protein levels may at least in part, explain the aerosolization behaviours of the various ST [36]. Tringe et al. [36] showed that fimbrial adhesin genes are up-regulated in air. It has been shown that *S. suis* possesses pili that have a putative role as adhesins [16]. The ST1 strains express the Sfp1 pilus but not the Sgp1 (Sfp1+/Sgp1−) [21]. It can be suggested that virulence factors could be involved in the preferential aerosolization process. Suilysin and extracellular factor are two virulence factors expressed by ST1 strains and not by ST25 and ST28 strains [21, 37]. Ye et al. demonstrated that the ST1 strains evolved from the ST25 strains and that they acquired 132 genomic islands, including 5 pathogenicity islands and 4 ST1 specific genes [19]. One or several genes acquired by ST1 isolates could favour bacterial aerosolization. Very recently, Atanassov et al. [38] identified nine proteins that differentiate ST1, ST25 and ST28 from other STs; including two that were overexpressed by ST1. These two proteins have not been purified for identification. Again, these proteins overexpressed by ST1 could promote the aerosolization of these strains. The proteins and genes specific or overexpressed by the ST1 strains could modify the water/air interface cell affinity or change the cell density. These factors could contribute to the preferential aerosolization process. The construction of mutants deficient in specific factors may allow evaluating the above hypotheses.

Strains less present in the air could be carried within the larger droplets which sediment. The preferentially aerosolized strains could be included in the smaller droplets that remained longer in the air and included in the air samples. It could explain the difference between air ratio of the different strains.

A better understanding of the aerosolization process of *S. suis* is essential to reduce the economic losses for the swine industry and to increase the swine’s health. Furthermore, aerosolization studies are of particular interest because *S. suis* is an important zoonotic agent especially in the Asian countries where the proximity between swine and farmers is more important.

## Conclusion

This study is a proof of concept and provides new evidence on the potential risks associated with the transmission of *S. suis* serotype 2 through bioaerosols. It also suggests a preferential aerosolization of *S. suis* serotype 2 ST1 strains and that preferential aerosolization of *S. suis* is likely a strain-dependent process, although more stains should be studied. This study emphases the importance to develop an exposure prevention strategy to protect the swine and the swine producers against *S. suis* infections.
